# A Review of Dynamic Mechanical Behavior and the Constitutive Models of Aluminum Matrix Composites

**DOI:** 10.3390/ma17081879

**Published:** 2024-04-18

**Authors:** Siyun Li, Tian Luo, Zhenlong Chao, Longtao Jiang, Huimin Han, Bingzhuo Han, Shanqi Du, Mingqi Liu

**Affiliations:** 1 School of Materials Science and Engineering, Harbin Institute of Technology, Harbin 150001, China; llsy@stu.hit.edu.cn (S.L.); luotian1452@gmail.com (T.L.);; 2 State Key Laboratory of Advanced Welding and Joining, Harbin Institute of Technology, Harbin 150001, China

**Keywords:** aluminum matrix composites, dynamic properties, strain rate, damage mechanism, constitutive model

## Abstract

Aluminum matrix composites (AMMCs) have demonstrated substantial potential in the realm of armor protection due to their favorable properties, including low density, high specific stiffness, and high specific strength. These composites are widely employed as structural components and frequently encounter high strain rate loading conditions, including explosions and penetrations during service. And it is crucial to note that under dynamic conditions, these composites exhibit distinct mechanical properties and failure mechanisms compared to static conditions. Therefore, a thorough investigation into the dynamic mechanical behavior of aluminum matrix composites and precise constitutive equations are imperative to advance their application in armor protection. This review aims to explore the mechanical properties, strengthening the mechanism and deformation damage mechanism of AMMCs under high strain rate. To facilitate a comprehensive understanding, various constitutive equations are explored, including phenomenological constitutive equations, those with physical significance, and those based on artificial neural networks. This article provides a critical review of the reported work in this field, aiming to analyze the main challenges and future development directions of aluminum matrix composites in the field of protection.

## 1. Introduction

With the rapidly growing demand for lightweight materials, aluminum and its alloys are increasingly used in aerospace, automobile and military applications due to their low density and high plasticity [1]. However, most metals have low strain hardening during plastic deformation and perform poorly in applications such as fretting, wear, impact, and energy absorption. How to improve various comprehensive indicators at the same time is a bottleneck in their development, which makes people shift the research focus from monolithic to composite materials. Among metal matrix composites, aluminum matrix composites (AMMCs) are regarded as the most promising structural materials due to their high specific strength, specific stiffness, enhanced tribological performance and low density [2]. AMMCs are a new type of material formed by combining aluminum-based metals with one or more reinforcing materials. While not compromising the good plasticity and toughness of the metals, composites also incorporate various excellent properties of the reinforcements. Compared to matrix alloys, composites offer a higher strength-to-weight ratio, specific stiffness, outstanding high-temperature mechanical properties, and excellent wear resistance, making them more widely used in industries such as aerospace, military, electronics, and transportation [3].

Different series of aluminum have been utilized in the manufacturing of AMMCs, including 2XXX, 6XXX, and 7XXX series, among others. The alloying elements like copper, magnesium, zinc, etc., are incorporated into pure aluminum to achieve distinct characteristics, rendering AMMCs suitable for a diverse array of applications [4]. For example, AMMCs based on 2XXX series aluminum alloys are renowned for their high strength and heat resistance, primarily used in the aerospace. AMMCs composed of 6XXX series aluminum alloys exhibit excellent machinability and weldability, finding widespread application in the automotive industry. Meanwhile, AMMCs of the 7XXX series are extensively utilized in the armor field due to their high strength and heat resistance [1,5].

Common reinforcements in aluminum matrix composites include carbides (SiC, B_4_C, TiC), nitrides (AlN, TiN, Si_3_N_4_), oxides (Al_3_O_2_, SiO_2_) and intermetallic compounds. Their forms can be particles, fibers, and whiskers, etc. The physical properties of commonly used reinforcements in AMMCs are given in Table 1 [6,7]. By changing the type, volume fraction, and morphology of the reinforcements, the performance of the composites can be changed and applied to required fields [8]. For example, boron carbide, silicon carbide, and aluminum oxide particles (or fibers) can form a very uniform bond with aluminum alloys, developing an attractive engineering material for high-strength, high-hardness, and wear-resistant applications [9].

Existing research shows that the performance of the same type of AMMCs are not only affected by factors such as the particle size and volume fraction of reinforcement, but the processing routes also play a key role in optimizing material properties. Traditional methods for manufacturing aluminum-based composites include powder metallurgy, pressure infiltration, stir casting, etc. [4]. Due to the poor wettability between the reinforcement and matrix, there is a heterogeneous distribution of reinforcement which can lead to clusters [10]. In recent years, laser additive manufacturing (LAM) has emerged as a novel manufacturing method, offering new possibilities for the fabrication of AMMCs. Compared to traditional manufacturing methods, additive manufacturing can produce unconventional structural components with high density, precision, and performance. Additionally, the layer-by-layer stacking of materials during the manufacturing process enables a homogeneous distribution of reinforcement and improves the wettability between the reinforcement and matrix. Although the AMMCs produced by additive manufacturing technology are still far from widespread commercial application, they have greatly promoted the research and application of new AMMCs [1,11].

Currently, particle-reinforced AMMCs demonstrate excellent overall performance, cost-effective production, well-established processes, and a bright future for development [3]. The AMMCs reinforced with low-particle content (<30 vol%) find applications in the aerospace and automobile industry, whereas those reinforced with high-particle content (>40 vol%) are utilized in armor protection, ballistic resistance, etc. In defense applications, ceramics such as B_4_C, SiC, and Al_2_O_3_ are commonly used as reinforcements in AMMCs due to their high hardness and low density. As a result, this review is dedicated to exploring the current research status of particle-reinforced aluminum matrix composites.

Compared with other metal matrix composites, AMMCs have wider application prospects in aerospace, armor protection, civil engineering and other fields due to their lightweight, high-specific strength and specific stiffness [4,12]. During the service process, AMMCs are inevitably subjected to various dynamic loads, such as aircraft landing, space debris impact, bird strikes and other high-strain-rate loading conditions [13]. At this time, the strain rate of the materials can reach 10^2^~10^4^ s^−1^ or higher, and the mechanical properties exhibited by the materials vary significantly under different strain rates. At high strain rates, AMMCs undergo large plastic deformations in a short time, accompanied by instantaneous and local high temperatures, exhibiting complex stress flow behaviors. Unlike static loading conditions, AMMCs subjected to dynamic impact typically exhibit higher flow stress due to strain hardening and strain rate hardening effects. Existing research indicates that the strain rate effect and strain rate sensitivity (SRS) of AMMCs under dynamic loads are more complex compared to static loading. Yield strength and flow stress exhibit varying trends with changes in strain rate. The deformation strain rate plays a significant role in influencing the macroscopic mechanical properties and microstructural evolution of the material [14]. For example, because the heat converted from plastic work in materials under dynamic impact cannot be conducted out in a short time, it is easy to form high-temperature areas in local areas, transforming from isothermal conditions to adiabatic conditions, forming special failure structures such as adiabatic shear bands [15]. Therefore, it is crucial to explore the dynamic mechanical behaviors of AMMCs and establish an accurate dynamic constitutive model in order to provide reliable basis for the material’s engineering applications [14].

This article reviews the research progress on the dynamic mechanical properties of AMMCs, introduces the plastic deformation behavior of materials under high strain rates from the two aspects of strengthening mechanisms and softening mechanisms, further discusses the research progress of several typical dynamic constitutive models, and finally summarizes and prospects the shortcomings in the current research on the dynamic performance of AMMCs under high strain rates.

## 2. Dynamic Mechanical Behavior of Aluminum Matrix Composites

According to the different strain rates, the loading conditions of materials can be divided into the following categories: (1) Creep: Refers to the situation where the strain rate of the material is below 10^−5^ s^−1^ during loading, and the influence of the strain rate on the mechanical properties of the material can be neglected. (2) Quasi-static: At this point, the strain rate of the material is between 10^−5^~10^−2^ s^−1^ and the effect of the strain rate on the material is also very small. (3) Intermediate: At this stage, the strain rate of the material is higher than 10^−2^ s^−1^, entering the range of dynamic loads, and the strain rate begins to affect the mechanical properties of the material. (4) High-speed (strain rate between 10^2^~10^4^ s^−1^). (5) Ultra-high-speed (strain rate above 10^4^ s^−1^). As the strain rate increases, the strain rate effect on the material under load becomes more pronounced [16,17].

In the early 19th century, research primarily focused on the mechanical properties of metals under static and low strain-rate-loading conditions, with limited understanding of the dynamic behavior of materials under high strain rates. Subsequently, the demands of war, such as impact loading and explosive shock, prompted research into the dynamic response of metals. It was discovered that the mechanical performance response of materials under external loading conditions primarily depends on the type and nature of the load, the strain rate of the loading, and the temperature during loading [16,18]. During this period, several methods for testing the dynamic mechanical behavior of materials under high-speed loading conditions were developed.

**Figure 1:**
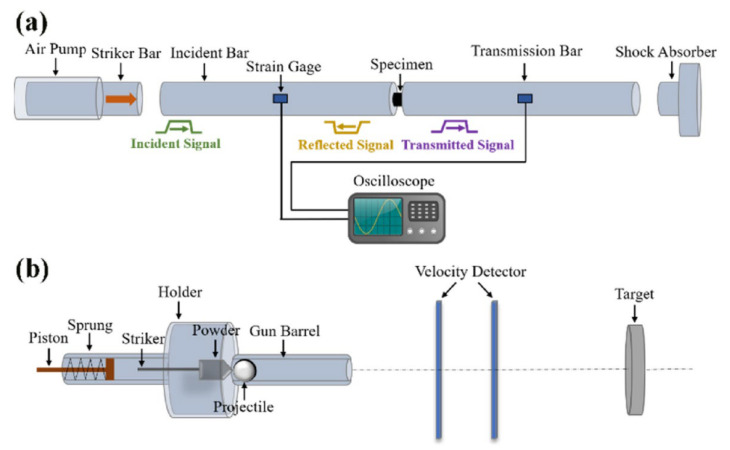
The SHPB system for dynamic compression testing. (**a**) Illustration of the SHPB apparatus; (**b**) powder gun apparatus [21].

Hopkinson [19] was the first to discover the relationship between the performance of steel and loading rate. He noted that material performance under impact loading varies from that under static loading, and invented the Hopkinson pressure bar to study the stress waveforms generated by ballistic impact and explosion. After that, Kolsky [20] improved this device, divided the pressure bar into two sections, and placed the sample in it, thereby successfully decoupling the stress wave effect and strain rate effect, and establishing the stress–strain relationship of materials under high strain rates. Because this device adopts a separate structure, it is called split Hopkinson pressure bar, abbreviated as SHPB. This device has been continuously improved by later generations and has become one of the most commonly used devices for testing the dynamic mechanical behavior of materials. As shown in the Figure 1 [21], the SHPB apparatus consists of an incident bar and a transmission bar with the specimen placed between them. The impact rod is propelled by compressed gas to strike the sample at high speed, forming an incident pulse in the incident rod and loading it onto the sample. After the sample is subjected to the incident pulse, it generates a reflected pulse that goes back and a transmitted pulse that passes through the transmission rod. According to the strain gauges attached to the rod, the signals of the incident wave, reflected wave, and transmitted wave are collected. After data processing, the loading strain rate and stress–strain data are obtained. The specific calculation formula is as follows [22]:

(1) $$\sigma \left(t\right)=E\frac{A}{A_{0}}\varepsilon_{T}(t)$$

(2) $$\dot{\varepsilon }\left(t\right)=-\frac{2C}{L}\varepsilon_{R}(t)$$

(3) $$\varepsilon \left(t\right)=-\frac{2C}{L}\int_{0}^{t}\varepsilon_{R}(t)dt$$

In the formulas, $\sigma \left(t\right)$, $\dot{\varepsilon }\left(t\right)$, $\varepsilon \left(t\right)$ represent stress, strain rate, and strain, respectively, *E* is the elastic modulus of the bars. *A*, *A_0_* are the cross-sectional areas of the bars and specimen, and *C* is the elastic wave propagation velocity in the bars, $\varepsilon_{R}$ and $\varepsilon_{T}$ denote the reflected and transmitted strain, respectively.

**Figure 2:**
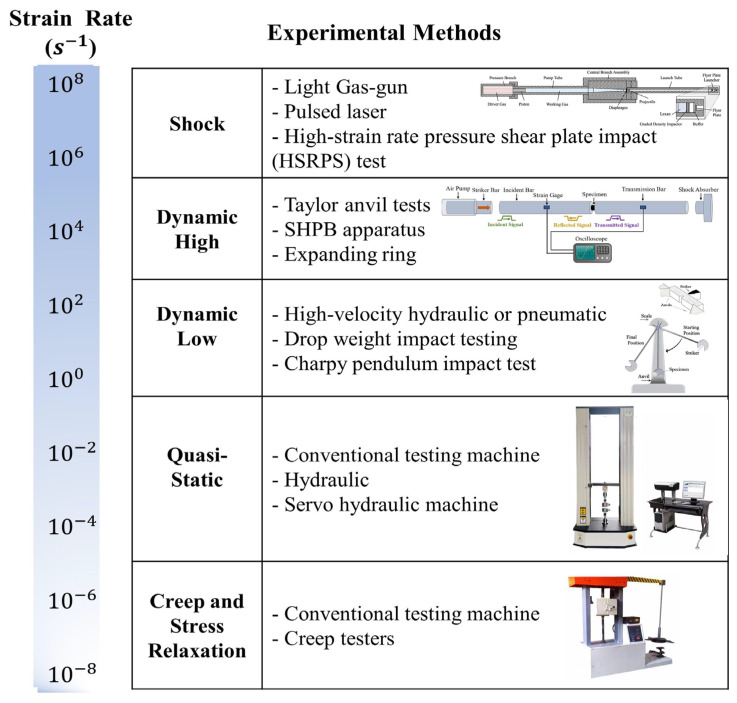
Testing methods and their achievable strain rates [21].

Subsequently, several dynamic experimental methods were developed, including the Taylor impact test, Charpy pendulum impact test, and ballistic resistance test. The Taylor impact test, proposed by Taylor in 1948 [23], is a method used to measure the dynamic mechanical properties of metals. Typically, it involves impact loading, where a specimen is subjected to a high-speed impact force by an impactor to induce plastic deformation and fracture. Compared to the SHPB, the Taylor impact test is simpler and more effective, especially under complex testing conditions such as high strain rates (10^4^~10^7^ s^−1^), large deformations, and elevated temperatures. However, data processing for the Taylor impact test is more complex, and sample processing is difficult. The Charpy pendulum impact test was initially proposed by the French scientist Charpy [24]. In this test, a V-shaped pendulum is raised to a certain height and then released, striking the middle portion of the specimen as it falls. This method simulates the stress state of the material under impact and measures its toughness by evaluating the impact energy absorbed by the material before fracture. The Ballistic resistance test is a method used to study the penetration behavior of materials under high-speed ballistic impact. This test typically involves shooting projectiles or bullets at the tested material using a launcher to simulate ballistic impact conditions. The Figure 2 [21] shows the commonly used testing methods and their applicable strain rate ranges. Currently, due to its widespread applicability, high-speed impact testing capabilities, simple data processing, and controllable experimental conditions, the SHPB device has become the mainstream method in the field of dynamic performance testing [25].

With the continuous development of research techniques in material dynamic mechanics, scholars have delved deeper into the study of the dynamic mechanical properties of aluminum matrix composites [26]. The plastic flow behavior of materials during high-speed plastic deformation is complex, and different types of aluminum matrix composites show different degrees of strain rate sensitivity. The dynamic response mechanisms of AMMCs predominantly exhibit strain hardening and strain rate hardening at intermediate strain rates. As the strain rate further increases, the AMMCs display dynamic recovery, dynamic recrystallization, and damage-induced softening [27,28]. In addition, because the heat generated by the dissipation of mechanical energy in the material under quasi-static conditions dissipates quickly, the deformation process of the material is generally treated as an isothermal process. However, the instantaneous deformation process of the material under high strain rates is similar to an adiabatic process. The heat converted from the plastic work produced by the external force in the system cannot flow out in a short loading time, causing the material to produce a large plastic deformation, accompanied by a local adiabatic temperature rise. At this time, the plastic deformation process of the material is manifested as a coupling mechanism constrained by strain rate sensitivity and temperature sensitivity [29]. Currently, research on the dynamic mechanical behavior of AMMCs mainly focuses on the superposition and competition of three processes: strain hardening, strain rate hardening, and matrix softening caused by adiabatic temperature rise under dynamic loading [30].

Since the interface reaction and evolution of the microstructure of the reinforcing materials and matrix alloys in AMMCs under dynamic impact conditions are significantly different from those under static load conditions, it is necessary to conduct in-depth research on the deformation behavior of AMMCs under dynamic loads, which holds important theoretical research significance and engineering application value.

### 2.1. Strengthening Mechanisms

#### 2.1.1. Strain Hardening

**Figure 3:**
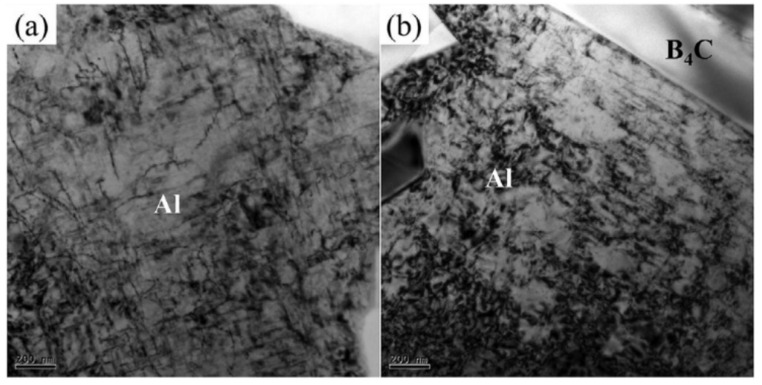
The images (**a**,**b**) showed the dislocations observed in the aluminum matrix of B_4_C/Al composites after high-speed impact [32]. Scare bar = 200 nm.

The dynamic mechanical behavior of aluminum matrix composites under impact loads has always been a hot topic in their development. Under impact loading conditions, materials are in a state of high pressure and high strain rate. At this time, strain and strain rate are the important factors affecting the dynamic response of materials. Existing research has shown that the strength of aluminum and its alloys increases with the increase in strain, and the true stress–strain curve of the material shows a gradually rising trend, demonstrating a significant strain hardening effect [31]. This is mainly due to the increase in strain, which increases the dislocation density in the material, thereby improving the strength of the material. As illustrated in the following Figure 3 [32], a significant number of dislocations can be observed in the aluminum matrix after high-speed impact [33]. However, due to the inclusion of reinforcements in AMMCs, phenomena such as particle damage, interfacial debonding, and adiabatic shearing occur under dynamic loads, exhibiting complex dynamic mechanical behavior [34,35].

Research has found that the strain hardening of aluminum matrix composites is not only related to the strain and strain rate during loading but also influenced by factors such as the type of matrix alloy, and the type and volume fraction of reinforcement. Rezayat et al. [36] explored the dynamic compressive properties of B_4_C/Al composite and the matrix alloy. They observed that, at low strains, the composite displayed a higher hardening rate, followed by a higher degree of softening before stress saturation. This is mainly due to the significant difference in the thermal expansion coefficients between the reinforcement and the alloy matrix at low strains, resulting in the generation of a large number of dislocations after heat treatment, further strengthening the material. However, as the strain increases, interface damage gradually occurs between the reinforcement and matrix. High-density dislocations concentrate at the corners of the B_4_C particles, causing stress concentration, leading to the cracking of brittle reinforcements, thereby reducing the load-bearing capacity of the material and resulting in strain softening. 

Ye et al. [37] studied the effect of SiC particle size on the dynamic compression performance of SiC/Al composites. The results indicated that, compared to quasi-static conditions, the composites exhibited strain hardening during initial deformation, with higher levels of hardening observed when the SiC particle size was smaller. Chen et al. [38] investigated the dynamic compression performance of B_4_C/6061Al composites with different volume fractions of B_4_C particles. The results showed that the strain-hardening capability of the material significantly increased with the increase in the volume fraction of B_4_C particles. However, as the strain further increased, the material exhibited strain softening, primarily due to the fragmentation of B_4_C particles and the debonding between B_4_C particles and the matrix.

**Figure 4:**
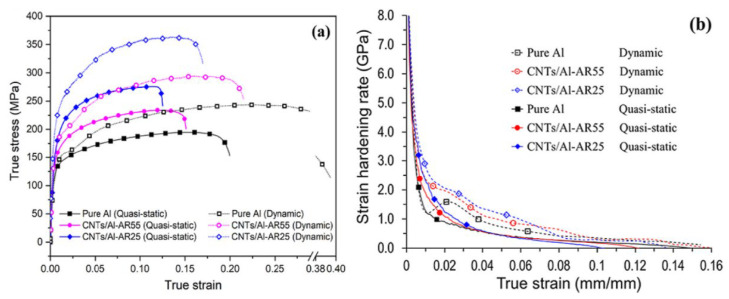
Typical mechanical characteristics of pure aluminum and CNTs/Al composites under various loading conditions. (**a**) True stress–strain curves and (**b**) the corresponding strain-hardening rate versus true strain [39].

Wang et al. [39] conducted dynamic and quasi-static tensile-compression tests on carbon nanotube-reinforced aluminum matrix composites (CNTs/Al). As shown in the Figure 4 [39], both pure aluminum and CNTs/Al exhibited stronger strain-hardening capability under dynamic loading conditions, where stress increases with increasing strain. Moreover, with the increase in strain rate, the strain-hardening capability of both materials significantly improved. Although the adiabatic temperature rise during high strain rate loading will cause a certain degree of thermal softening, the strain-hardening capability of the materials is markedly enhanced compared to static loading conditions. This analysis suggests that the strain-hardening capability of the material is associated with the dynamic recovery rate of dislocations during plastic deformation. As the strain rate increases, the dynamic recovery of the material is inhibited, leading to enhanced dislocation multiplication, resulting in a macroscopic increase in strain-hardening capability. Additionally, when subjected to external loading, the matrix deforming through strain coordination generates a large number of dislocations around the reinforcements, leading to CNTs/Al exhibiting higher strain-hardening capability compared to pure aluminum.

The analysis above indicates that, in the early stages of the dynamic deformation of AMMCs, the increase in strain leads to the aggregation and multiplication of dislocations inside the matrix. Due to the pinning effect of the reinforcement on dislocations and the increase in geometrically necessary dislocations (GND) resulting from the thermal mismatch between reinforcements and the matrix [40], aluminum matrix composites generally exhibit a higher strain-hardening rate compared to static conditions. However, as the strain further increases, the intensified interface damage between the reinforcement and the matrix promotes the dynamic recovery of the matrix, resulting in some materials undergoing strain softening. The critical point of strain softening depends on factors such as the type of material, size, and volume fraction of the reinforcement.

#### 2.1.2. Strain Rate Hardening and Strain Rate Sensitivity

Material deformation behavior is influenced by the strain rate during loading, causing changes in yield strength, flow stress, and failure strain. This phenomenon is known as strain rate sensitivity (SRS). Aluminum matrix composites typically exhibit higher flow stress and plastic strain under dynamic impact compared to static loading, demonstrating strain rate hardening. Moreover, the hardening capability varies among different composites, depending on factors such as the type of reinforcement, volume fraction, aspect ratio, and strain rate [41,42].

**Figure 5:**
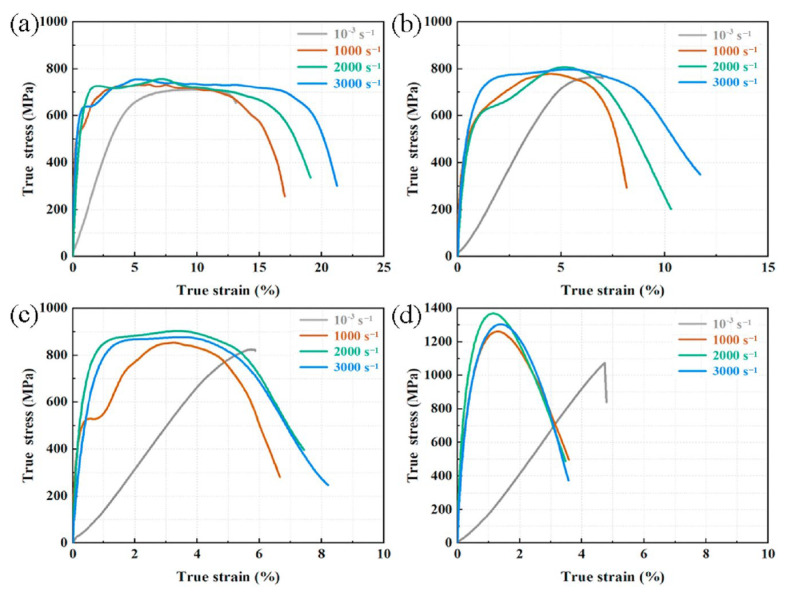
Quasi-static and dynamic stress–strain curves of composites with varying B_4_C content. (**a**) 20 vol% B_4_C/Al; (**b**) 30 vol% B_4_C/Al; (**c**) 40 vol% B_4_C/Al; (**d**) 50 vol% B_4_C/Al [43].

Li and Ramesh [41] studied the effect of SiC particle (SiC_p_) volume fraction and aspect ratio on the mechanical properties of SiC_p_/Al under high strain rate. They found a significant strain rate hardening effect, which increased with the volume fraction of SiC particles. The study also showed that the strain rate correlation is not only related to the aspect ratio of the particles, but also to the particle shape (spherical or cylindrical). Bao Lin [42], based on an axially symmetric crystal cell model simulation, discovered that the strain rate hardening effect of the material is related to the volume fraction of particles. Due to the constraint effect of the particles on dislocations, the strain rate hardening effect of the composite material is significantly higher than that of the matrix. Wang et al. [43,44] manufactured 2024Al/20–50vol.%B_4_C composites with a lamellar-interpenetrated structure, and studied their quasi-static and dynamic properties at strain rates from 0.001 to 3000 s^−1^. As shown in the Figure 5 [43], before the strain rate increased to 3000 s^−1^, the compressive strength of the composites increased with the strain rate, demonstrating strain rate hardening. Additionally, owing to the formation of a rigid network structure in composites with high B_4_C content, the strength of the material also increased with the increase in reinforcements. 

**Figure 6:**
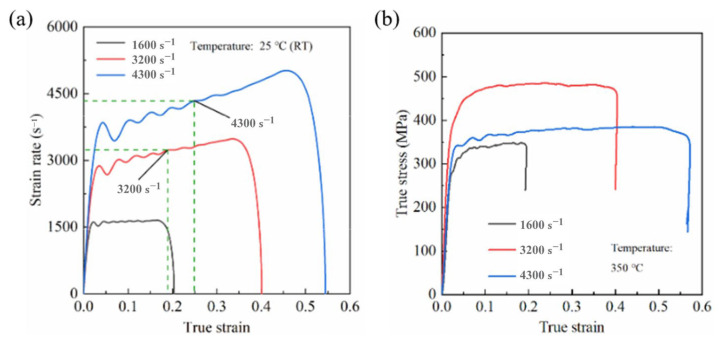
Stress–strain curves of SiC/Al at different temperatures and strain rates. (**a**) True stress–strain curves at room temperature (RT); (**b**) true stress–strain curves at 350 °C [46].

Lee et al. [45] prepared Al-based composite materials reinforced with SiC particles of different sizes using pressure infiltration and found that the dynamic compressive strength of the specimens was higher than the quasi-static strength. According to the strain rate hardening effect, as the strain rate increased, the flow stress of the material further increased. Additionally, under dynamic compression, the Al matrix partially melted due to adiabatic heating, leading to increased deformation of the matrix, which effectively inhibited crack propagation and increased the strain. Suo et al. [46] studied the dynamic compressive mechanical properties of SiC/6092Al at different temperatures. As shown in the Figure 6 [46], their findings revealed notable strain rate hardening at room temperature, showing an escalation in flow stress and yield strength as the strain rate increased. However, when the temperature reached 350 °C, the material experienced strain rate softening. The proportion of fractured particles decreased, while the proportion of particles experiencing interface debonding increased, indicating that higher temperatures promoted the melting of the aluminum matrix and the formation of adiabatic shear bands. At this point, strain rate hardening was overcome by thermal softening.

The above studies indicate that the majority of AMMCs exhibit strain rate hardening under dynamic loading, with the hardening effect being more pronounced compared to the basic alloy. However, for high-volume fraction composites or under high-temperature conditions, strain rate softening can also occur due to the fracture of reinforcement particles and the melting of the aluminum matrix. This means that, as the strain rate increases, the material’s flow stress decreases.

In recent years, a considerable amount of research has been conducted on the strain rate sensitivity of AMMCs under dynamic loading. The above discussion also indicates that, under high strain rate dynamic loading, there exists a correlation between the flow stress and strain rate of composites, demonstrating strain rate sensitivity (SRS) [47]. The material’s strain rate sensitivity coefficient represents the degree of sensitivity of stress to strain rate, and its expression is as follows [48]:

(4) $$\sum =\frac{\sigma_{d}-\sigma_{s}}{\sigma_{s}}\frac{1}{ln\;({\dot{\varepsilon }}_{d}/{\dot{\varepsilon }}_{s})}$$

where $\sigma_{s}$ and $\sigma_{d}$ represent the flow stresses under quasi-static and dynamic loads at a constant plastic strain, respectively; $\dot{\sigma_{s}}$ and $\dot{\sigma_{d}}$ represent the corresponding strain rates for quasi-static and dynamic loads, respectively.

Existing studies indicate that the strain rate sensitivity of aluminum matrix composites strongly depends on the strain rate sensitivity of the matrix alloy. If the matrix alloy is sensitive to strain rate, then the composites also exhibit strain rate sensitivity [39]. However, the strain rate sensitivity of composites is more complex. The yield strength and flow stress also vary with factors such as the volume fraction and type of reinforcement. Additionally, most composites exhibit different levels of strain rate sensitivity within a certain range of strain rates [49].

Xie et al. [50] studied the dynamic performance of SiC_p_/Al and found that when the volume fraction of SiC is low, the composite is not sensitive to strain rate. However, when the volume fraction of SiC increases to 50%, the material’s yield strength shows a positive correlation with strain rate, exhibiting significant strain rate sensitivity. Wang et al. [43] studied the dynamic compression performance of B_4_C/Al composite materials with four volume fractions (20%, 30%, 40%, 50%). Research indicated that, as the content of B_4_C increases in composites, the strain rate sensitivity (SRS) of the material becomes more pronounced, especially when the content of B_4_C exceeds 40%. However, with the increase in strain rate, the SRS of the material initially increased and then decreased. This phenomenon is largely attributed to the flow localization and voids evolution induced by ASBs during compression.

**Figure 7:**
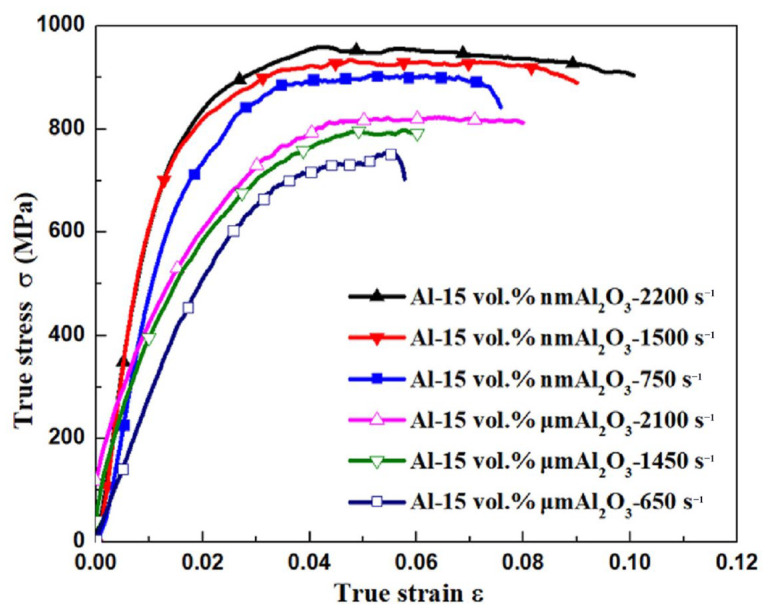
Dynamic compressive stress–strain curves of composites under different strain rates and with different sizes of reinforcement [52].

Behm et al. [34] conducted quasi-static and dynamic behavior analysis of nano- or semi-micro-sized B_4_C/Al. They found that the SRS of the composite under dynamic loading depends on the matrix alloy, and the strain rate sensitivity of composites containing B_4_C reinforcements of different sizes varies. Wang et al. [51] fabricated B_4_C/2024Al composites with different sizes of B_4_C particles using squeeze casting and investigated the compressive strength of the composites at high strain rates using a split Hopkinson pressure bar setup. Their research results show that the dynamic compressive strength of fine particle-reinforced composites is higher than that of coarse particle-reinforced composites, and the sensitivity to strain rate is more significant; in addition, compressive strength exhibits strain rate insensitivity or even negative sensitivity due to damage to B_4_C particles under high strain rates, resulting in strain softening in the composites. Using high-energy ball milling and hot pressing, Liu et al. [52] prepared micron-scale Al_2_O_3_-reinforced (Al-MMC) and nano-scale Al_2_O_3_-reinforced aluminum matrix composites (Al-MMNC) to investigate the influence of reinforcement particle size on mechanical properties. Both composites show a strain rate hardening effect, which increases with increasing reinforcement volume fraction, as depicted in the Figure 7 [52]. However, at the same volume fraction, the strain rate sensitivity of Al-MMNC was lower than that of Al-MMC, which may be attributed to differences in strengthening mechanisms.

In addition, temperature also has a significant influence on the strain rate sensitivity of materials. Suo et al. [46] investigated the high-temperature dynamic compressive performance of powder metallurgy-manufactured 30 vol.% SiC/6092Al composites under three different environmental temperatures and strain rates. The experimental results indicate that, at room temperature, the flow stress of the composites is essentially insensitive to the strain rate. However, as the temperature increases, the composites begin to exhibit different strain rate-dependent behaviors, with the rheological stress showing nonlinear changes with increasing temperature. Additionally, scanning images of the samples suggest that both environmental temperature and strain rate have significant effects on the microscale failure mechanisms of the composites. With increasing temperature, the proportion of failure particles decreases, and there are significant changes in the primary failure modes of the particles. Perng et al. [27] conducted tensile experiments on Al_2_O_3_/6061Al composites with different Al_2_O_3_ content under various strain rates using a Hopkinson tension bar, investigating the factors influencing the strain rate sensitivity of the composites. The results showed that the addition of reinforcement particles contributes to enhancing the strain rate sensitivity of the composites. The strain rate sensitivity of the ultimate tensile strength increases with temperature. Rezayat et al. [36] prepared B_4_C-reinforced Al-Mg alloy, and investigated the dynamic deformation of composites and an Al-Mg alloy at high temperatures. The results indicated that, compared to the Al-Mg alloy, composites exhibit a higher hardening rate. However, after the flow stress reaches the peak stress, the material exhibits dynamic softening. Moreover, an increase in the volume fraction or size of the reinforcement particles, as well as an increase in temperature, promote softening. Huang et al. [53] tested the dynamic performance of 14 vol.% SiC/2014Al over a range of strain rates from 0.001 to 1 s^−1^ and temperature from 355 to 495 °C. The results showed that the flow stress of the material increased with increasing strain rate at different temperatures. The strain rate sensitivity and temperature sensitivity maps indicated that temperature had a more significant impact on deformation mechanisms compared to the strain rate.

From the above, it can be known that due to the restriction of the reinforcement on the plastic flow of the matrix and the high dislocation accumulation rate around the reinforcement during the deformation process, the strain rate sensitivity of composites is often higher than the matrix [54]. The main reasons why composites exhibit greater strain rate sensitivity than matrix materials can be attributed to three factors: (1) the mismatch in plastic strain between the matrix and reinforcement leads to an increase in the geometrically necessary dislocation density corresponding to deformation coordination; (2) the resistance of the reinforcement to dislocation motion at high strain rates leads to an increase in internal stress in the matrix, resulting in a substructure strengthening effect; (3) the constraint of reinforcement particles on the matrix material leads to a significantly higher actual strain rate of the local matrix than the macroscopic strain rate, and the thermal activation mechanism in the high strain rate region causes the material to exhibit high strain rate sensitivity [55,56]. In addition, with the change of parameters such as the content and size of the reinforcement, the strain rate sensitivity of the composites will also change, and the temperature during the loading process of the material will also have a great impact on the dynamic mechanical properties.

### 2.2. Softening Mechanisms

During the dynamic loading deformation process, due to the synergistic effect of strain hardening and strain rate hardening, the AMMCs will show higher yield strength and flow stress than quasi-static conditions. However, when strain or strain rate is further increased, some materials may undergo strain softening, where the flow stress of the material gradually decreases with increasing strain, exhibiting a distinct difference from the dynamic stress–strain curve of aluminum alloys. Existing studies indicate that damage to the reinforcement and interfacial debonding during dynamic loading can lead to the strain softening of AMMCs [43,44]. Additionally, the softening of the aluminum matrix at high strain rates or temperatures, as well as the formation of adiabatic shear bands, can counteract the work-hardening effect, reducing the flow stress level of the composite material and causing strain softening [46]. This work will review the strain softening of composites from the perspectives of the cracking of reinforcement particles and matrix softening.

#### 2.2.1. Cracking of Reinforcement Particles

When subjected to impact loads, due to the fact that the reinforcements in the composites are mostly ceramics, which are much more brittle compared to the matrix alloy, the stress concentration at the sharp corners of the reinforcement under high-speed impact cannot be relaxed by the deformation of the matrix, eventually forming cracks on the surface and inside of the particles. When the concentrated stress exceeds the shear strength of the particles, the particles crack. The fracture of the particles will reduce the effective bearing area, thus weakening the strengthening effect of reinforcement [57,58]. In addition, due to the difference in the thermal expansion coefficients between the reinforcement particles and the matrix, this deformation incompatibility will cause voids at the particle-matrix interface. As the voids develop and accumulate, interface debonding eventually occurs. These damages affect the dynamic compressive properties of AMMCs by weakening the strengthening effect of reinforcement [53].

**Figure 8:**
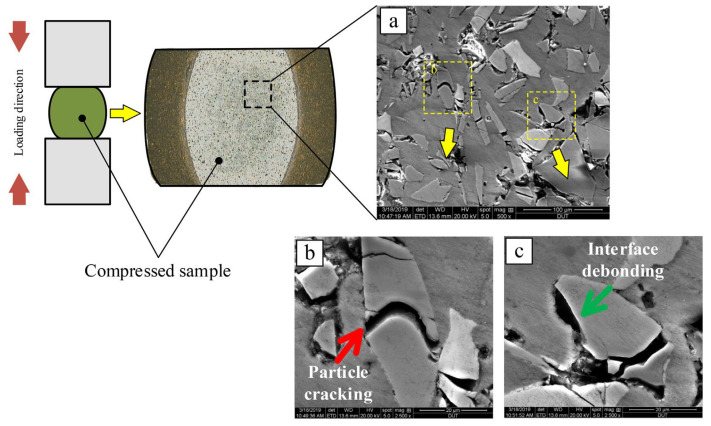
The cross-section of the SiC/Al sample and fracture of SiC particles after dynamic compression. (**a**) Cross-section of the SiC/Al sample, (**b**) particle cracking, (**c**) interfacial debonding [59].

Most researchers [35,59] suggest that under high strain rates, the fracture of reinforcement particles in composites can be divided into three types: cracked particles, shattered particles, and interfacial debonding with the matrix. The Figure 8 [59] illustrates cracked particles and particles debonding from the matrix.

Lee et al. [35,60] used the SHPB setup to investigate the dynamic deformation behavior of high-volume fraction SiC_p_/Al composites and analyzed the microstructural changes occurring during dynamic deformation. They observed three damage modes in the dynamic deformation process: particle fracture, interface debonding, and matrix softening. Among them, the particle cracking and interfacial debonding between the reinforcement and the matrix are major parameters affecting the dynamic compressive behavior. Chen et al. [38] compared the microstructure of B_4_C/6061Al with different volume fractions after compression and found that the composites with a high content of B_4_C particles are more likely to form microcracks after dynamic loading because, as the content of B_4_C particles increases, the micropores in the material increase, and interface debonding is more likely to occur. In addition, some researchers have found [61,62] that a larger proportion of fractured reinforcement particles occur in areas where particle aggregation is present. This suggests that there is a greater residual stress–strain concentration in the particle accumulation area under high strain rate compression.

**Figure 9:**
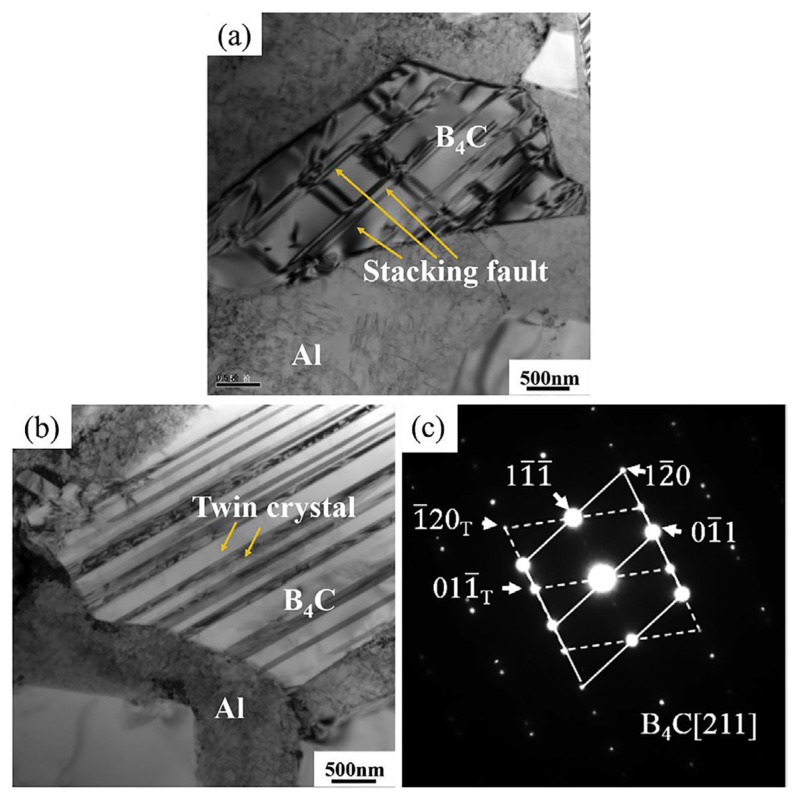
High-speed impact causing high-density stacking faults (**a**) and micro-twins (**b**,**c**) in B_4_C/Al composites [32].

Jo et al. [63] investigated the dynamic performance of AMMCs reinforced with different types of ceramic particles. They found that, at lower strains, some coarse ceramic particles fractured while finer particles remained mostly intact. As deformation progressed, interface debonding between the reinforcement and matrix began to occur, and the particles with interface debonding accounted for the largest proportion in the final damaged structure. The authors also observed that the dynamic performance of AMMCs was dependent on the type of reinforcement ceramics when the volume fraction of reinforcements was constant. Specifically, composites reinforced with hybrid particles exhibited higher dynamic compressive strength than those with single-type reinforcement. Wu et al. [64] found that during compression deformation of 15 vol.% SiC_p_/Al composites, with the increase in strain rate, the deformation type of the material transitioned from uniform deformation to localized deformation. This led to an increase in the degree of particle fracture, and larger-sized, multi-angular reinforcement particles were more prone to fracture during compression due to higher local stresses and more defects. Chao et al. [32] investigated the ballistic behavior and microstructure evolution of B_4_C/AA2024 composites. The results indicated that the density of dislocations generated near the edge of the B_4_C particles was higher than that in their interior. As shown in Figure 9a,b [32], a relatively high density of stacking faults and micro-twins can be observed within the B_4_C particles after high-speed impact. Additionally, Figure 9c indicates that the twin plane is ($1,\bar{1},\bar{1}$).

It can be seen that particle-reinforced AMMCs typically undergo particle fracture and interfacial debonding under dynamic loading conditions. The location and degree of particle fracture are not only related to the type, size and shape of the particles, but also to the aggregation of particles, the strain and strain rate during loading. As the number of broken particles in the composites increases, the load-bearing function of the particle skeleton structure decreases. The failed particles will become the source of cracks causing the destruction of the composites, further inducing low-stress brittle fracture, thereby reducing the load-bearing capacity of the material and causing strain softening [63].

#### 2.2.2. Matrix Softening

1. dynamic recovery and recrystallization

In addition to accelerating atomic transitions, which will intensify the plastic flow, two common softening mechanisms, dynamic recovery (DRV) and dynamic recrystallization (DRX), may also take place in the matrix alloy when AMMCs experience plastic deformation under dynamic loads. For face-centered cubic (FCC) metals with more slip systems, twinning will also occur at high strain rates [65]. These three microscopic mechanisms change the properties of composites by affecting the microstructure of the matrix [28,66]. Under impact load, as the strain rate increases, the dislocation density in the material increases, and the interweaving of dislocations forms local distortion, which promotes the occurrence of DRV. Subsequently, DRV changes the distribution of point defects and dislocations in the matrix. The spatial rearrangement annihilates dislocations, thereby weakening the work hardening caused by the accumulation and entanglement between dislocations. Under a certain strain, the work hardening rate and the dynamic recovery rate will gradually reach a dynamic balance. When the strain hardening and dynamic recovery can no longer store more fixed dislocations, the dynamic recrystallization mechanism starts [15,67].

**Figure 10:**
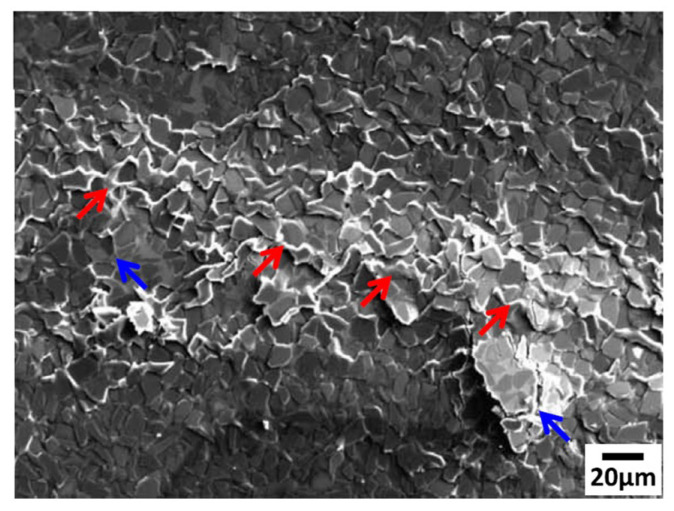
The red arrows indicated the molten Al matrix near the specimen surface, while the blue arrows represented fracture of intermetallic compounds [60].

Lee et al. [60] investigated the dynamic compression properties of SiC_p_/A356 composites and found that near the surface of the directly impacted sample, a part of the Al matrix is easily melted due to a temperature rise higher than the melting temperature of the A356 Al alloy, as shown by the red arrow in the Figure 10 [60]. The molten matrix alloy effectively prevents crack propagation and increases compressive strain.

**Figure 11:**
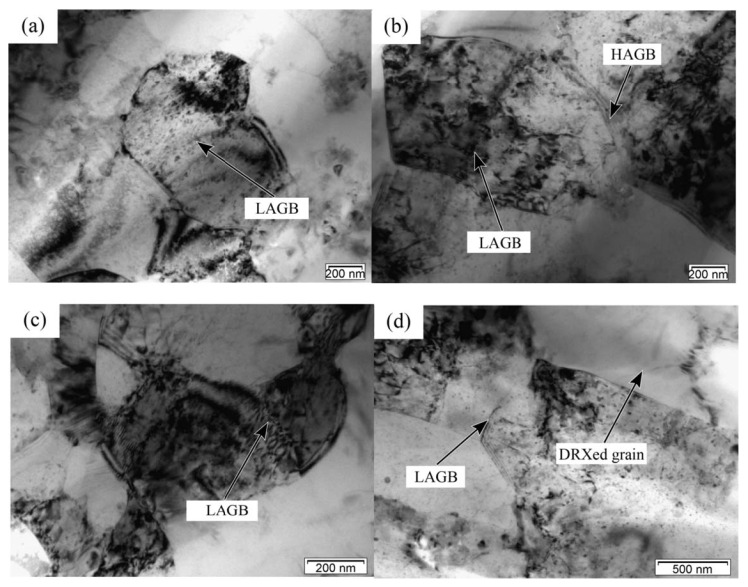
TEM images of SiC/Al after compression at different strain rates at 793 K. (**a**) 0.1 s^−1^; (**b**) 1 s^−1^; (**c**) 5 s^−1^; (**d**) 10 s^−1^ [68].

Wang et al. [68] investigated the thermal softening mechanism of low-volume fraction nano-SiC reinforced aluminum matrix composites during compression and explored the impact of thermal deformation conditions on the dynamic softening mechanism. Research results show that high-density dislocations will be generated inside the material at low temperatures and high strain rates. At this time, the cross slip of dislocation is the main softening mechanism. When the temperature and strain rate increase, the dislocation density inside the matrix decreases, and new grains without distortion are observed, indicating that the DRV and DRX are the main softening mechanisms of the composite, as shown in the Figure 11 [68].

Tang et al. [69] conducted dynamic uniaxial compression tests on 17 vol.% SiC_p_/7055Al composites to study the influence of deformation parameters on the flow stress of the composites. The experimental temperature was 250~450 °C. The results show that when the temperature is between 250 and 350 °C, the composite exhibits typical DRV characteristics, but when the temperature further increases, the softening mechanism of the composite changes to DRX.

Sun et al. [59] studied the effects of particle damage and thermal softening on the dynamic compression behavior of SiC_p_/Al composites and found that the flow stress of the composites decreased with increasing temperature at a constant high strain rate. This change pattern is consistent with the Al matrix. Due to the increase in temperature and the adiabatic temperature rise inside the matrix at high strain rates, the Al matrix undergoes DRV and DRX, resulting in softening. Thermal softening increases the flow properties of the matrix, further reducing the strengthening effect of the particles. The study points out that the dynamic compression performance of composites at high temperature results from the interplay between particle reinforcement and matrix thermal softening, defining it as particle–heat coupling. Based on the above studies, the dominant mechanism of Al matrix softening behavior is related to strain rate and temperature. Most researchers [70,71] will establish the relationship between the dominant mechanism and the Zener-Hollomon parameter Z as follows:

(5) $$Z=\dot{\varepsilon }exp(\frac{Q}{RT})$$

where $\dot{\varepsilon }$ represents strain rate (s^−1^); *Q* denotes the activation energy of hot deformation; *R* stands for universal gas constant (8.314 J mol^−1^ K^−1^); and *T* represents absolute temperature (K).

It is generally believed that the smaller the Z value, the more conducive it is to the occurrence of DRX, and the size of the generated dynamic recrystallization grains decreases as the Z value increases [72,73]. For materials with high stacking fault energy such as aluminum alloys, because the stacking fault band between two partial dislocations is narrow, it is easy to gather into full dislocations, so the dislocations are more likely to cross-slip and climb. Usually, at room temperature, DRV occurs, and DRX occurs only during thermal deformation [74].

**Figure 12:**
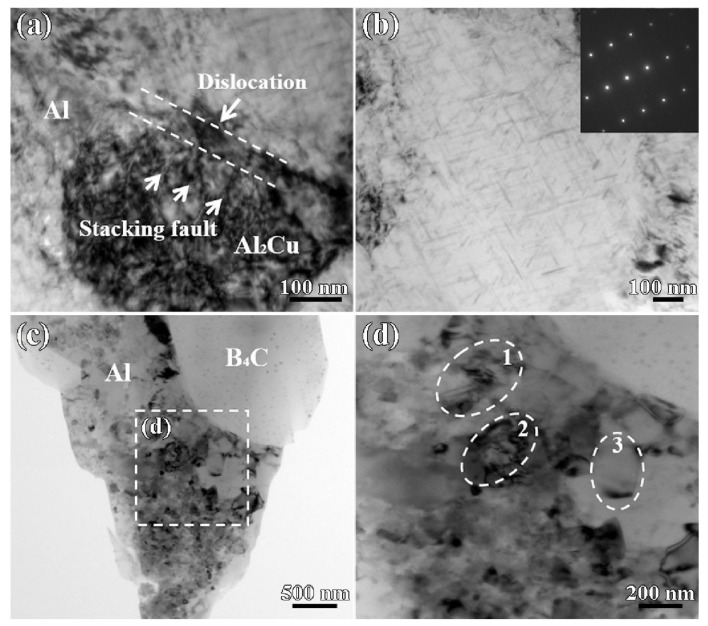
TEM images of 20 vol% B_4_C/Al after impact at a strain rate of 3000 s^−1^: (**a**) stacking faults and dislocations in Al_2_Cu particle; (**b**) precipitate phases; (**c**) recrystallized grains in the Al matrix; (**d**) local magnification of (**c**), and encircle the recrystallized grains as 1–3 [43].

Chao [56] indicated that there is a significant difference in the sensitivity of AMMCs to temperature under quasi-static and dynamic loads. The occurrence time of DRV and DRX based on dislocation movement will decrease with an increase in temperature. Therefore, when the temperature rises, the softening of the material is more significant, leading to a sharp decrease in strength. In addition, Wang et al. [43] investigated the quasi-static and dynamic properties of 20–50 vol.%B_4_C/2024Al composites at various strain rates (10^−3^~3 × 10^3^ s^−1^). The results indicate that, with increasing strain and strain rate, the dislocation density in the Al matrix increases, providing the driving force for DRV and DRX within the alloy. As shown in Figure 12d, the continuous increase in dislocation leads to grain elongation, eventually developing into recrystallized grains without distortion.

2. adiabatic shear band

Adiabatic shear refers to the process in which most of the plastic deformation energy conversion into heat in the material during high-speed deformation cannot diffuse out in time due to the short duration (usually around 100 µm), leading to a sharp rise in local temperature, causing a significant decrease in the deformation resistance of the alloy and triggering the failure process [75]. When studying the dynamic mechanical behavior of metallic materials, adiabatic shear phenomena are often one of the issues of concern for researchers because the formation of adiabatic shear bands (ASBs) is often a precursor to the fracture failure of materials during dynamic impact processes. Its basic characteristics include the observation of ASBs at the microscale, the occurrence of thermal plastic instability on the macroscopic stress–strain curve, and the high-speed deformation process approaching adiabatic conditions. The width of the ASBs is 1–200 µm, and their formation is related to the following three factors: large shear strain, high strain rate (above 10^3^ s^−1^), and high temperature [76,77].

Zener et al. [78] first discovered the phenomenon of adiabatic shear in the study of the plastic deformation process of iron, and proposed that the band-shaped deformation zone formed in the high-speed deformation process of the material is the adiabatic shear band. Subsequently, Cho et al. [79] also found the formation of adiabatic shear bands in SiC whisker (SiC_w_)-reinforced 2124Al composites after ballistic impact. Hanina et al. [80] proposed the localization of an adiabatic shear, conducted systematic research on the adiabatic shear, and divided the shear bands into two types: deformation bands and phase transformation bands. The research shows that when the material is under high strain rate and ultra-high-speed impact, it mainly forms an adiabatic shear band dominated by phase transformation bands.

For metals such as aluminum, iron, copper, titanium, and their alloys, the adiabatic temperature rise generated under high strain rate impact can be calculated using the following equation [81]:

(6) $$∆T=\int_{0}^{\varepsilon_{f}}\frac{\beta \sigma }{\rho C_{m}}d\varepsilon$$

where β represents the rate of plastic deformation energy converted into heat; $\varepsilon_{f}$ is the failure strain; ρ is the density and $C_{m}$ is the specific heat capacity.

Zhu et al. [82] used the equation to calculate the adiabatic temperature rise under high strain rate compression for high-volume fraction TiB_2_/Al composites and found it was only 15~20 K, which is far from enough for the formation of a molten Al matrix. Chao [74] proposed that the premise of using this equation to calculate ∆T of AMMCs is to regard the composite as a homogeneous material, but in the actual deformation process, the reinforcement particles are relatively brittle and difficult to deform; at this time, the deformation of the material is almost fully completed by the ductile matrix, so the local temperature rise in the composite is actually much higher than the result calculated in the equation. The above equation is more suitable for low-volume fraction composites.

Research has shown [83,84] that metal matrix composites are more prone to adiabatic shear failure under dynamic loading impacts compared to the matrix alloy. The content and size of the reinforcement have a significant impact on the formation of adiabatic shear bands. Behm et al. [34] studied the quasi-static and dynamic mechanical properties of B_4_C/5083Al composites with different particle sizes. They found that both sizes of B_4_C-reinforced composites exhibited non-uniform deformation under high strain-rate compression, forming adiabatic shear deformation bands. Within these shear bands, local plastic deformation of the Al matrix and initiation of crack bands were observed, while such phenomena were not observed in the unreinforced 5083Al alloy. Dai et al. [84] proposed that the smaller the particles of the reinforcement, the greater the possibility of forming adiabatic shear bands in AMMCs. Xu et al. [85] also found this phenomenon. They studied the effect of the size of the reinforcement particles in SiC_p_/Al composites on the localization of material shear, and found that the composites reinforced with the smallest SiC particles (3 µm) formed a clear shear band, while the composites with larger reinforcement particle sizes of 17 µm and 37 µm tended to deform non-uniformly, and no visible shear bands were found. This indicates that ASB is more likely to form in composites reinforced with smaller particles.

**Figure 13:**
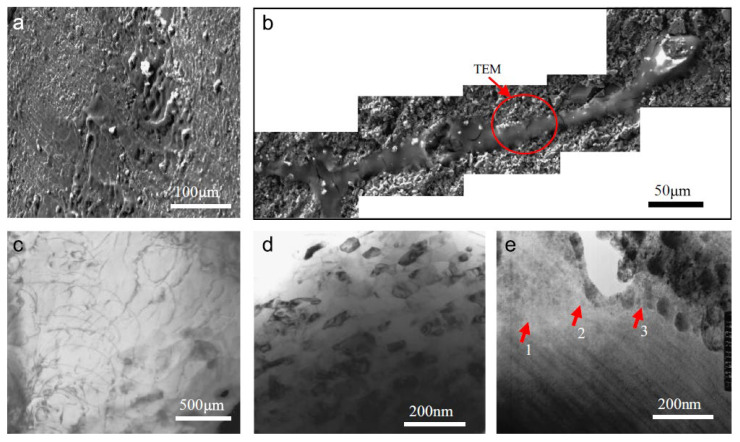
SEM and TEM images of the adiabatic shear bands: (**a**) fluid-like bands; (**b**) narrow bands; (**c**) dislocations; (**d**) fine grains (less than 100 nm); and (**e**) amorphous and nano-crystalline grains as 1–3 [86].

Zhu et al. [86] investigated a shear band characterized as the melting phase transformation of TiB_2_/Al composites. They analyzed the adiabatic shear failure and microstructural characteristics of shear bands by the SEM, TEM and HREM. As shown in the Figure 13 [86], the formation of ASB was caused by localized adiabatic temperature rise. Abundant dislocations and fine grains were present at the edges of the shear bands, while a significant amount of amorphous, nanocrystalline, and ultrafine grains were found within the band. The authors believed that the heat generated by the plastic deformation work of the material during high-speed deformation would cause a large temperature non-uniformity, which would affect the formation of the microstructure near the shear band, and the failure of the material would also be caused by shear localization.

**Figure 14:**
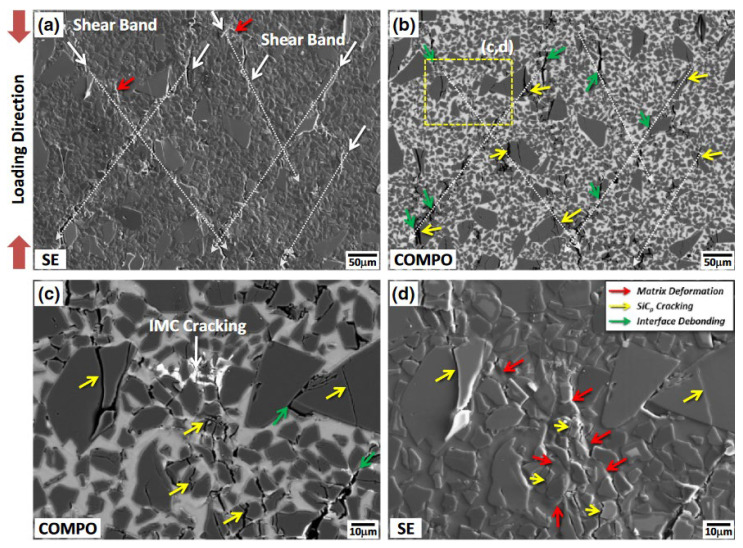
From (**a**–**d**) are SEM images of SiC/7075Al after dynamic compression at a strain rate of 2800 s^−1^, showing adiabatic shear bands in the Al matrix, the cracking of SiC particles and interface debonding [45].

Lee et al. [45] studied the effect of strain rate on the dynamic compression properties and damage structure of SiC_p_/7075Al. The Figure 14 [45] shows the SEM image of the side of the sample after compression at a strain rate of 2800 s^−1^, in which the white arrow indicates the adiabatic shear zone, the yellow arrow indicates the cracking of SiC particles (SiC_P_), and the green arrow indicates the interface debonding between the reinforcement and the matrix. It can be seen that, compared with static load, the local deformation area of the material under dynamic load forms an adiabatic shear band, and the molten Al matrix can be observed around it. The cracking and local matrix deformation of particles mostly occur near the shear band, which means that deformation mainly occurs in local areas. Zhu et al. [87] used Al_35_Ti_15_Cu_10_Mn_20_Cr_20_ high-entropy alloy particles as the reinforcement of 2024Al, and analyzed the microscopic damage characteristics and adiabatic shear failure mechanism of composites under dynamic load impact. They found that the local deformation and adiabatic shear cracks of composites develop gradually. As the strain rate and reinforcement volume fraction increase, the deformation characteristics of the material change to localized deformation. Both parameters can promote the formation of adiabatic shear bands in the matrix, causing instability and cracking damage in the composites.

According to the above research, the deformation process of aluminum matrix composites under dynamic loading is caused by the coupling of multiple mechanisms, including the strengthening effects caused by strain and strain rate hardening, as well as the fragmentation of reinforcement particles, the DRV of the aluminum matrix, softening due to DRX and the formation of ASBs [59]. In the early stage of material deformation, the work-hardening effect is much greater than the softening effect, which is manifested as an increase in the macroscopic stress–strain curve. As the deformation proceeds, the internal dislocation density of the material increases, prompting the fragmentation of particles and the DRV and DRX of the matrix. At this time, the softening effect is greater than the work-hardening effect; the stress of the material gradually decreases with an increase in strain, until the continuous accumulation of plastic deformation leads to an adiabatic temperature rise inside the matrix, and finally the material undergoes adiabatic shear instability failure [28].

## 3. Dynamic Constitutive Model of Aluminum Matrix Composites

The constitutive model aims to predict the change of stress with strain under certain deformation conditions of the material. Phenomena such as strain hardening, dynamic recovery, and the dynamic recrystallization of the material during the deformation process are closely related to the strain ε, strain rate $\dot{\varepsilon }$, temperature *T*, etc. [88]. Therefore, the constitutive relationship of a material describes the relationship between stress *σ* and factors such as ε, $\dot{\varepsilon }$, *T* during the deformation process. Its general form can be expressed as follows:

(7) $$\sigma =\varphi \left(\varepsilon ,\dot{\varepsilon },T\right)$$

The study of the dynamic behavior of AMMCs originated from the study of the plastic flow behavior of metallic materials under high strain-rate loading. This behavior has different rate–temperature coupling properties and microscopic mechanisms under static loading. The design of material structure and performance evaluation in aerospace, casting molding, armor protection and other fields require a large number of dynamic load experiments and numerical simulations. At this time, establishing an accurate material-dynamic constitutive model is the basis and key to the reliability of structural numerical simulations [89]. Moreover, the constitutive model can also break through the limitations of experimental conditions and play an important role in studying the dynamic mechanical response of materials under higher strain-rate conditions. Currently, researchers have conducted extensive studies on the constitutive models of metals, proposing different constitutive models from different perspectives. The dynamic constitutive model of AMMCs is based on these models and appropriately modified to accurately predict the plastic deformation behavior of different composites by considering the special effects of structure and microstructure. Currently, the research on constitutive models of AMMCs reported can be mainly summarized into the following three types: (1) phenomenological-based models, which correlate empirical observations with mathematical functions to predict flow stress; (2) physical constitutive models established based on the micromechanical method of plastic deformation mechanism, which are rooted in plastic deformation mechanisms, and start from microstructural features such as dislocation, grain structure, twinning, etc.; (3) and artificial neural network (ANN) constitutive model, leveraging machine learning for flow stress prediction [90,91].

### 3.1. Phenomenological Constitutive Model

Presently, a variety of constitutive models are employed to characterize the mechanical properties of metal materials subjected to dynamic loads. Among them, phenomenological constitutive models, with their simple forms and intuitive rules, were first applied in the field of engineering [90]. The phenomenological constitutive model is based on experimental data and utilizes techniques such as data fitting to determine the influence of strain, strain rate, and temperature on the dynamic mechanical behaviors of materials. Among them, the Johnson–Cook model, which is the most widely used in the field of elastic–plastic impact calculations, is a representative of this type of constitutive model [74,92].

The J-C constitutive model was established by Johnson et al. [93] in 1963. It is used to describe the flow behavior of metal materials under large deformation, high strain rate, and high-temperature conditions. Due to its simple form, it has been widely applied and embedded in commercial finite element software such as ABAQUS 6.8 and ANSYS 5.4 [94]. The J-C constitutive relationship considers the equivalent flow stress of the metal as the product of three components: strain function, strain rate function, and temperature function. Its specific form is as follows [95]:

(8) $$\sigma =(A+B\varepsilon^{n})(1+Cln{\dot{\varepsilon }}^{*})(1-T^{*m})$$

Here, σ is equivalent stress, and equivalent plastic strain is ε; ${\dot{\varepsilon }}^{*}=\dot{\varepsilon }/{\dot{\varepsilon }}_{0}$ is the dimensionless plastic strain rate, where $\dot{\varepsilon }$ is the plastic strain rate, and ${\dot{\varepsilon }}_{0}$ is the reference strain rate; $T^{*}=(T-T_{r})/(T_{m}-T_{r})$ is the dimensionless temperature, where *T* represents the absolute temperature, $T_{m}$ represents the melting point of the material, and $T_{r}$ is room temperature; *A*, *B*, *n*, *C* and *m* are material constants, where *A* is the yield stress at the reference temperature and reference strain rate, while *B* represents the strain-hardening coefficient, *n* is the strain-hardening index, *C* and *m* represent the material constants of the strain-rate hardening coefficient and the thermal softening coefficient, respectively. $\left(A+B\varepsilon^{n}\right)$, $\left(1+Cln{\dot{\varepsilon }}^{*}\right)$, $\left(1-T^{*m}\right)$ describe the strain-strengthening effect, strain-rate strengthening effect and thermal softening effect of the material, respectively.

The J-C constitutive model can achieve relatively high accuracy when describing metal materials, but the properties of AMMCs are between pure metals and pure reinforcements, and change with the volume fraction of reinforcement. There are still some problems in the dynamic behavior of materials, including the inability to describe the strain softening caused by the accumulation of material damage [59], insufficient accuracy in describing medium- and high-volume aluminum matrix composites, the inability to realize temperature effects in a large temperature range, and the inability to describe the sudden change in stress caused by dynamic recrystallization or other phase changes of the material. In order to solve these problems, researchers modified the J-C model [96].

Zhan et al. [97] proposed a rate-dependent PD model to modify the influence of the strain rate on matrix properties in the J-C model, and analyzed the mechanical properties and the fracturing behaviors of particle-reinforced MMCs. Chao et al. [74] believed that the particle damage that causes composite softening and the related mechanisms of aluminum matrix softening are related to the strain during the dynamic loading process of the material, so they introduced a decreasing function including strain to describe this softening phenomenon. They used the modified J-C constitutive equation to describe the dynamic compression behavior of 25 vol.% and 47 vol.% B_4_C/2024Al at different temperatures. The fitting results indicate that the modified J-C model predicts less error compared to the original J-C model, providing a more accurate description of the dynamic behavior of B_4_C/2024Al composites.

Niu et al. [98] modified the J-C model based on the Eshelby equivalent inclusion model and the Weibull model [99]. They introduced a tangent function tanh(*ε*) as a softening term to correct the impact of particle damage at different deformation degrees, and introduced a Gaussian function to correct the thermal softening of the material under adiabatic temperature rise. The final corrected J-C constitutive equation is as follows:

(9) $$\sigma =\left(A+B\varepsilon^{n}\right)\left(1+Cln{\dot{\varepsilon }}^{*}\right)\{aexp[-\left(\frac{T^{*}-m_{1}}{m_{2}}{)}^{2}\right]\}\{D+(1-D)[\tanh(\frac{1}{\frac{\varepsilon_{cr}}{\varepsilon_{c}}\varepsilon }{)}^{r}{]}^{s}\}$$

**Figure 15:**
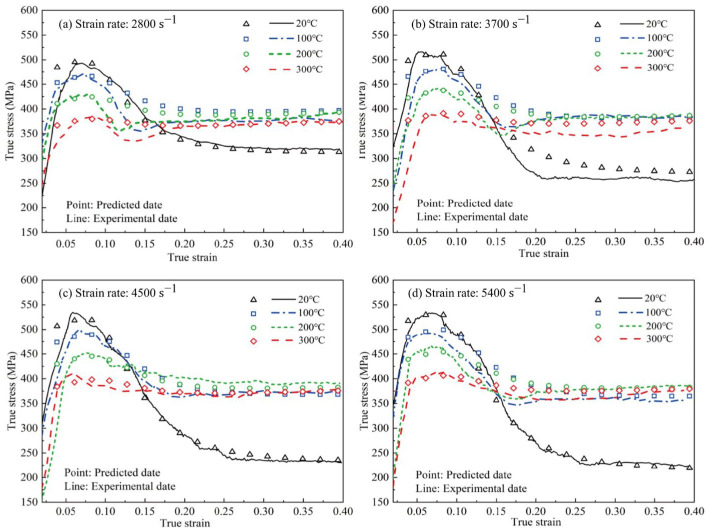
Comparison of the predicted results with the experimental results of the dynamic compressive properties of 50 wt% Si/Al composites [98].

As shown in the Figure 15 [98], the modified J-C constitutive model by the researchers exhibits good agreement with experimental results in the plastic deformation regime. Moreover, it maintains high precision even under conditions of large strain and high temperatures, indicating that the addition of thermal softening and damage softening terms can significantly improve the accuracy of the J-C model.

Considering the influence of phase transition, dislocation density, and material structure on flow stress during the material deformation process, some researchers [100,101,102] proposed the following J-C constitutive models to describe the dynamic mechanical behavior of metals:

(10) $$\sigma =\left(A_{1}+B_{1}\varepsilon +B_{2}\varepsilon^{2}\right)\left(1+Cln{\dot{\varepsilon }}^{*}\right)exp\;[(\lambda_{1}+\lambda_{2}ln{\dot{\varepsilon }}^{*})(T-T_{r})$$

Among them, $A_{1}$, $B_{1}$, $B_{2}$, C, $\lambda_{1}$, $\lambda_{2}$ are constants related to the material. Based on this form of the J-C constitutive model, Tang et al. [69] considered the comprehensive effects of thermal softening and strain hardening on flow stress, and proposed a modified model to describe the dynamic compression behavior of 17 vol.% SiC_p_/Al composites. The fitting results show that the accuracy of model prediction is greatly improved. In addition to the J-C constitutive model, commonly used phenomenological-based models include Li-Ramesh, Arrhenius, KH, KHL, etc. [41,103,104,105]. Among them, the Li-Ramesh model is also often used to describe the dynamic properties of aluminum matrix composites; its basic form is as follows:

(11) $$\sigma (f,\varepsilon ,\dot{\varepsilon })=\sigma_{0}(\varepsilon )g(f)[1+(\frac{\dot{\varepsilon }}{{\dot{\varepsilon }}_{0}}{)}^{m}][1+f(\frac{\dot{\varepsilon }}{{\dot{\varepsilon }}_{0}}{)}^{m}\;]$$

where f is the volume fraction of reinforcement particles; ε is strain; $\dot{\varepsilon }$ is strain rate; *m* is the strain rate-related parameter related to the matrix; $\sigma_{0}(\varepsilon )$ is the stress–strain curve of the matrix under quasi-static deformation; g(f) is the strengthening equation, which represents the relationship between the volume fraction of reinforcement and the yield stress under quasi-static conditions.

The Li-Ramesh model includes the matrix properties, particle content, particle shape, strain, and strain rate and other strengthening factors. It is accurate in predicting the dynamic performance of aluminum matrix composites with low-volume fractions, but it does not contain a thermal softening term, ignoring the effects of particle damage and thermal softening on the strengthening effect of composites during the deformation process, resulting in the lower prediction accuracy of flow stress under high strain rates and high particle content [28].

Sun et al. [59] considered the softening effect caused by particle damage and adiabatic temperature rise during the dynamic loading process, and combined the L-R model and J-C model to predict the dynamic mechanical behavior of 30 vol.% SiC_p_/Al composites. He believes that the impact of reinforcement particles on the material during the deformation process of the material can be expressed by the probability of SiC particle damage and the probability of interface debonding. By improving the L-R model, the following function is introduced to describe the impact of particle damage:

(12) $$S\cdot f[1-f_{de}-\left(1-f_{de}\right)f_{c_{r}}]$$

**Figure 16:**
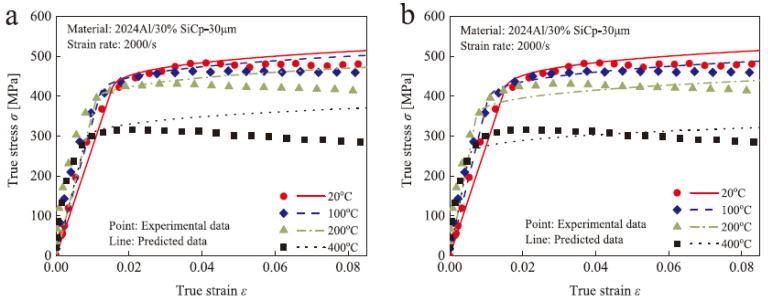
Comparison between the stress–strain curves of predicted data and experimental results. (**a**) The predicted results without considering particle–heat coupling; (**b**) the predicted results considering particle–heat coupling [59].

In the equation, *S* represents the reinforcement coefficient of SiC particles, f represents the volume fraction of SiC particles, $f_{de}$ represents the probability of particle–matrix interface debonding and $f_{c_{r}}$ represents the probability of particle cracking. The author further considered the adiabatic temperature rise caused by plastic deformation work during the dynamic compression process, modified the thermal softening term in the original J-C equation, and established a constitutive model with particle–thermal coupling. As shown in the Figure 16 [59], when considering the influence of thermal softening on the reinforcing effect of reinforcement particles, the model’s predictive accuracy significantly improves at high temperatures. This indicates that the coupling of the thermal softening function with the particle reinforcement function is crucial for the accuracy of the model in composites.

As shown in Table 2, many researchers have modified the J-C constitutive model to simulate the dynamic mechanical response of aluminum alloys or AMMCs under specific experimental conditions. Most modifications considered the influence of particle damage and thermal softening on flow stress by adjusting the thermal softening term in the original J-C constitutive model. The accuracy of the modified J-C model has significantly improved, but there are also some limitations. Since the model parameters are fitted through the dynamic mechanical behavior of a certain composite material under specific conditions, the model is not universal between different composites. In addition, the J-C constitutive model was originally used to describe the dynamic behavior of metals. The prediction accuracy for AMMCs with high-volume fraction is poor. At present, research on the dynamic behavior of AMMCs often uses different models according to the different contents of reinforcement, lacking a general J-C constitutive model that can accurately describe the dynamic performance of MMCs over a wide range of volume fractions, temperature, and strain rate [106].

### 3.2. Physical Constitutive Model

The physical constitutive model uses the knowledge of the physical deformation process to establish a constitutive model of equations, involving thermodynamic theory, thermal activation theory, dislocation slip, etc., and can analyze materials from microscopic deformation mechanisms [115,116]. The commonly used physical constitutive models include the ZA model, VA model, NN model, and MTS model [117]. Nevertheless, in comparison to phenomenological constitutive models, physical constitutive models typically exhibit greater complexity in their formulation, necessitating a larger number of material constants and experimental data to determine model parameters. As a result, their utilization in practical engineering applications is relatively limited.

The ZA constitutive model is one of the most widely used physical constitutive models. It was proposed by Zerilli and Armstrong [118] in 1987, considering the effects of strain hardening, strain rate strengthening, and thermal softening on the flow stress of metals, and dividing the flow stress into thermal activation term and non-thermal term. Due to the different effects of temperature and strain rate on face-centered cubic (FCC) and body-centered cubic (BCC) metals; for example, BCC metals show higher temperature sensitivity and strain rate sensitivity than FCC metals, so the forms of ZA constitutive models of FCC and BCC metals are also different. For FCC metals, dislocations must overcome the obstacle of dislocation forest, and the thermal activation area decreases with an increase in plastic strain; for BCC metals, dislocations mainly overcome the Peierls–Nabarro barrier, which makes its thermal activation behavior independent of plastic strain [15,113]. Therefore, the ZA models for FCC and BCC metals, respectively, can be obtained as follows:

(13) $$\sigma =C_{0}+C_{1}\varepsilon^{1/2}exp(-C_{3}T+C_{4}Tln\dot{\varepsilon })$$

(14) $$\sigma =C_{0}+C_{1}\exp\left(-C_{3}T+C_{4}Tln\dot{\varepsilon }\right)+C_{5}\varepsilon^{n}$$

where $C_{0}\sim C_{5}$ and n are fitting parameters. It can be observed that, for FCC metals, temperature and strain rate will affect the strain-hardening behavior. In response to the different plastic flow behaviors of various types of metals, many researchers have proposed modified models based on the ZA model to make it more widely applicable. Zhang et al. [119] believed that the parameters of the original ZA model were regarded as constants, which was not consistent with the actual situation. After considering the influence of strain, temperature and strain rate on the parameter C_3_ in the model, he modified the original ZA equation, and the final model can better describe the plastic flow behavior of alloy IC10. Samantaray et al. [120] further considered the effects of thermal softening, strain rate hardening, and isotropic hardening, and combined the characteristics of the J-C model to further modify the ZA model, which has been widely used to predict the high-temperature flow behavior of metals, as shown below:

(15) $$\sigma =(C_{1}+C_{2}\varepsilon^{n})exp[-\left(C_{3}+C_{4}\varepsilon \right)T^{*}+\left(C_{5}+C_{6}T^{*}\right)ln{\dot{\varepsilon }}^{*}]$$

Similarly to the above, $C_{0}\sim C_{5}$ and m are all fitting parameters. Based on this, Senthilkumar et al. [71] used the improved ZA model to predict the high-temperature deformation behavior of nano-SiC particle-reinforced 5083Al composites. They obtained the parameters of the ZA model by fitting stress–strain curves of the material at strain rates of 0.01~1 s^−1^, and the resulting ZA model accurately predicted the deformation behavior of the composites. Yuan et al. [121] used the modified ZA model, Arrhenius model, artificial neural network model, etc., to forecast the high-temperature deformation behavior of 15 vol.% SiC/Al composites. The results indicated that the predictions of these constitutive models were consistent with experimental results, but the strain rate and temperature had a significant impact on the predictions of the ZA model.

**Figure 17:**
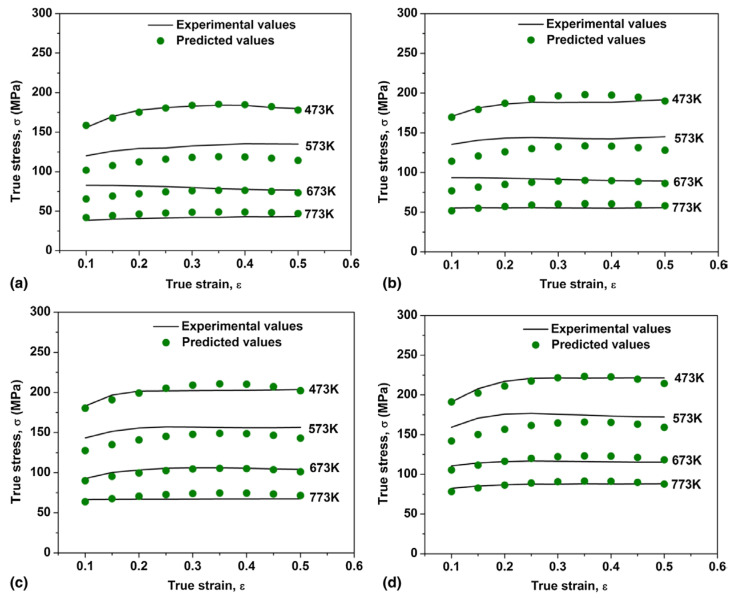
Comparison of the flow stress predicted by the modified Z-A constitutive model with experimental data at different temperatures and strain rates: (**a**) 0.01 s^−1^; (**b**) 0.1 s^−1^; (**c**) 1 s^−1^; (**d**) 10 s^−1^ [91].

Rudra et al. [91] predicted the thermal deformation behavior of SiC/5083Al using the J-C model and the ZA model modified by Samantaray [120]. They evaluated the applicability of the models by comparing the correlation coefficient, average absolute relative error, etc. As shown in the Figure 17 [91], the obtained ZA model fits well with the experimental data, and compared to the J-C constitutive equation, the modified ZA model exhibited higher prediction accuracy. Dalvand et al. [122] employed the J-C model, Zerilli-Armstrong (ZA) model, and Arrhenius model to simulate the thermal deformation process of micron SiC-reinforced AMMCs. Their study revealed that the classical models utilized for predicting the thermal deformation behavior of metal alloys can also effectively predict the behavior of AMMCs. However, it was noted that the ZA model is specifically applicable for simulating material-thermal deformation at elevated temperatures, yet its predictive accuracy was found to be inferior to that of the other two models. It is evident that there exists significant variation in the predictive accuracy of models for different AMMCs. Adjusting the models appropriately according to material characteristics can greatly enhance their accuracy.

In addition to the ZA model, there are several other physical constitutive models utilized for describing the thermal deformation of metals across various strain rates and temperatures. The VK model, developed by Voyiadjis et al. [123] based on the ZA model, utilizes the concept of thermal activation energy and the dislocation interaction mechanism to simulate the flow stress of metals, while also considering the influence of dislocation density evolution on material flow stress. On the other hand, the MTS model [124] considers mechanical threshold stress and demonstrates the proficient prediction of the hot deformation behavior of metals under high strain rates, albeit demanding extensive experimental data for parameter determination. At present, the construction of most physical constitutive models is relatively complex and difficult, which limits their wide application.

### 3.3. Artificial Neural Network Constitutive Model

In processes such as impact, explosion and hot forming, there are complex coupling relationships between strain rate, temperature, plastic strain and microstructure evolution. Therefore, it is quite difficult to establish a traditional constitutive model with wide application range, simple form and high accuracy [125]. An artificial neural network (ANN) is a system that uses computers to simulate the structural characteristics of biological brains to handle complex models such as multi-element and nonlinear models. Its biggest feature is that it does not need a clear algorithm to connect input and output data, but continuously corrects the model through data to reduce errors and predict unknown data. Therefore, for the establishment of constitutive models, artificial neural networks are not limited to fixed model functions, greatly improving the accuracy of the model, and can simplify the manual calculation process and improve the ability of the model to predict the results in unknown ranges [15,126]. Compared to traditional phenomenological and physical constitutive models, an artificial neural network (ANN) demonstrates enhanced adaptability and flexibility, expanding the application prospects for constructing constitutive models. However, it lacks consideration of the physical relationships between inputs and outputs, rendering it a black-box technique. Moreover, ANN relies heavily on the training database and cannot replace the theoretical significance and research value of traditional constitutive models [127].

**Figure 18:**
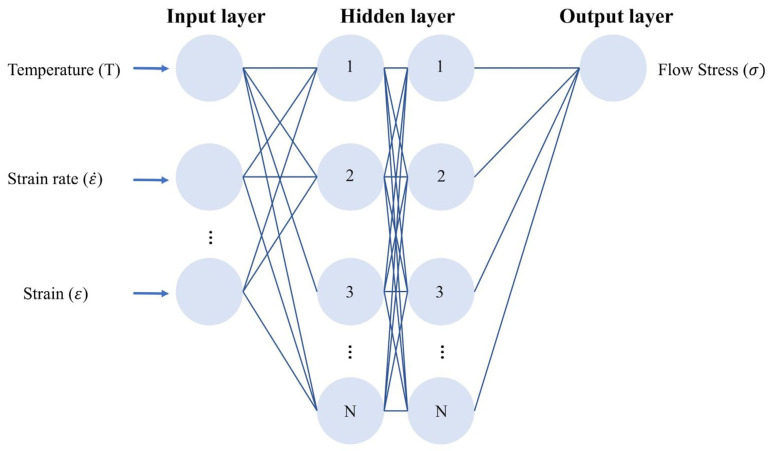
Schematic diagram of the BP ANN employed in the metal dynamic constitutive model [129].

Generally speaking, an ANN has an input layer, a hidden layer and an output layer. The number of nodes in the input layer is determined by the number of variables, while the number of nodes in the output layer is determined by the number of calculation results. The number of layers and nodes in the hidden layers needs to be optimized, which is crucial for improving the calculation accuracy of ANNs. Currently, the Backpropagation Algorithm (BP) is the most widely used multi-layer artificial neural network learning algorithm. Approximately 80% to 90% of the actual applications of ANNs use the BP algorithm or its modified forms. The output of its upper layer nodes is the input of the lower layer nodes, including feedforward and backpropagation algorithms [128]. The typical BP ANN results for metal dynamic constitutive models are illustrated in the Figure 18, where the input layer typically includes temperature (T), strain rate ($\dot{\varepsilon }$), and strain (ε), and the output layer represents the flow stress (σ) [129].

In 1995, Rao et al. [130] applied the four-layer BP neural network to the flow stress prediction of the thermal deformation process for the first time. They trained the neural network using the flow stress data of medium carbon steel at different temperatures and strain rates, successfully predicting results under other operating conditions using the trained model. Currently, ANN constitutive models have found extensive application in predicting the microstructure evolution and mechanical characteristics of materials during various thermal forming processes. Pandya et al. [131] utilized a modified J-C constitutive model and ANN model to predict the deformation behavior of 7075 aluminum alloy during processes like hot stamping. The results indicate that, compared to the J-C model, the trained ANN model can better predict the deformation behavior of the material after strain hardening. Jalham et al. [132] used an ANN constitutive model to study the influence of reinforcement content and process parameters on the thermal deformation behavior of Al_2_O_3_/Al composites. They compared the results with those predicted by an Arrhenius constitutive model. The experimental fitting data showed that the prediction accuracy of the ANN model was higher, and it was found that the reinforcement content and deformation conditions had a complex nonlinear effect on the flow stress. Senthilkumar et al. [71] also compared the accuracy of the thermal deformation process of the TiC/Al predicted by four constitutive models. The results showed that, compared with the phenomenological constitutive model and physical constitutive model used, the evaluation accuracy of the ANN model was higher.

Liu et al. [133] compared the predictive accuracy of the traditional Arrhenius constitutive model and the ANN model for the deformation behavior of (CNT_s_–Al)/ZA27 composites under high temperature. It is found that because the traditional Arrhenius equation can only describe stable flow processes, such as strain hardening, the DRV and DRX of material, there is a large difference between the prediction results and the experiment when unstable deformations such as micro-cracks and shear bands occur. However, the ANN model exhibits higher predictive accuracy, indicating that the ANN model can better predict the mechanical behavior of the composite. Smirnov et al. [126] studied the deformation behavior and softening mechanism of SiC/Al at temperatures of 300~500 °C and low strain rates. They linked microstructural parameters with thermomechanical action parameters on metals to train neural networks, thereby reducing the time required for training. Moreover, the neural networks they constructed can accurately describe the thermal deformation behavior of composites with high precision.

Currently, well-trained BP-ANN models can accurately predict the flow stress of metals, but their prediction accuracy deteriorates when data deviate from the training set. An increasing number of researchers are turning to backpropagation-deep neural networks (BP-DNNs) for forecasting unknown data. Cheng et al. [134] employed an improved Arrhenius model and the DNN model to predict the thermal deformation behavior of ZA270.15Ce alloy at temperatures ranging from 200 to 320 °C and strain rates from 0.01 to 10 s^−1^, revealing that the DNN model exhibits higher prediction accuracy.

In contrast to conventional constitutive models, artificial neural network models are more accessible and provide superior predictive accuracy. Nevertheless, their utilization in forecasting the dynamic mechanical behavior of AMMCs is somewhat restricted. It will take some time and effort to establish the ANN model that can accurately describe the dynamic behavior of various types of aluminum matrix composites.

## 4. Conclusions

Aluminum matrix composites have been widely used in aerospace, civil engineering, armor protection and other fields due to their excellent properties. The performance of AMMCs is affected by the fabrication process, the type of aluminum matrix and reinforcement, etc. The traditional manufacturing methods limit the application of AMMCs due to some shortcomings. In recent years, additive manufacturing has provided greater possibilities for the application of new AMMCs. During service, AMMCs inevitably undergo various dynamic loads. The research on their dynamic mechanical properties and deformation mechanisms has always been a focus and challenge in this field. This article summarizes the plastic deformation behavior of AMMCs under high strain rates, focusing on its strengthening and softening mechanisms. Furthermore, the currently commonly used dynamic constitutive models of AMMCs are summarized. The main outcomes could be summarized as follows:

1. The deformation process of the composites under dynamic loading is caused by the coupling of multiple mechanisms. Due to the interaction between the matrix, reinforcement and IMCs, the mechanical behavior of the AMMCs during dynamic loading has significant differences compared with quasi-static;
2. In the early stage of the dynamic deformation of AMMCs, the material is mainly affected by the strengthening mechanisms, showing strain hardening and strain rate hardening. As strain and strain rate further increase, the reduction in flow stress due to particle damage and matrix softening in AMMCs leads to adiabatic shear instability, resulting in strain softening;
3. The dynamic constitutive models currently used to describe the deformation behavior of AMMCs can be divided into three aspects: phenomenological constitutive models, physical constitutive models, and artificial neural network constitutive models: the traditional phenomenological constitutive models have been widely used because of their simple form, but they lack sufficient understanding of the physical mechanism, so they cannot accurately describe the relationship between material plastic deformation and microstructure evolution; the physical constitutive models take into account the heat transfer of the AMMCs during the deformation process, but its construction process requires the solution of complex material microscopic and thermodynamic performance parameters, which limits its application range; the constitutive models based on artificial neural networks that have emerged in recent years has better prediction accuracy and flexibility, but there are few studies on the application of numerical simulation of plastic forming, and it will take some time to establish a universal dynamic mechanical model that can accurately describe various types of aluminum matrix composites;
4. Current research is mostly focused on AMMCs with medium/low-volume fractions, and there is less research on the dynamic deformation behavior of Al matrix composites with high-volume fraction (>50 vol%). Further research is needed to establish the destruction criteria and constitutive equations of the material. In the future, research on the dynamic mechanical behavior of high-volume fraction AMMCs should be strengthened, and consideration should be given to establishing a general constitutive model that can accurately describe the dynamic performance of AMMCs in a wide volume fraction, wide temperature, and wide strain rate range.

## Figures and Tables

**Table 1:** Physical properties of commonly used reinforcements in aluminum matrix composites [6,7].

Properties	B_4_C	SiC	Al_2_O_3_	TiC	TiB_2_
Density (g/cm^3^)	2.5–2.52	3.12–3.2	3.6–3.98	4.93	4.52
Modulus of elasticity (GPa)	450–470	400–450	300–450	400	550
Hardness, Vickers (GPa)	29–35	22–23	12–18	24–32	21–23
Fracture toughness (MPa·m^1/2^)	2.5–3.5	4.0–4.6	3.5–4.0	3.2–6.7	6–8

**Table 2:** Summary of the current modified J-C constitutive models.

Applicable for	Characteristic	Equations	Reference
7050-T7451 aluminum alloy	Considered the coupling effect of strain and strain rate.	$\sigma =(A+B\varepsilon^{n})(1+C(\varepsilon ,\dot{\varepsilon })ln{\dot{\varepsilon }}^{*})(1-T^{*m})$	[107]
Al-4.8Cu-1.2Mg alloy	Modified the strain hardening (n) and strain rate hardening (C) coefficients.	$\sigma =(A+B\varepsilon^{f\left(\varepsilon ,\dot{\varepsilon }\right)})(1+f(\dot{\varepsilon })(\varepsilon ,\dot{\varepsilon })ln{\dot{\varepsilon }}^{*})(1-T^{*m})$	[108]
6061 aluminum alloy	Coupled the temperature term in the original J-C model with the strain rate.	$\sigma =\left(A+B\varepsilon^{n}\right)\left(1+Cln{\dot{\varepsilon }}^{*}\right)exp[\left(\lambda_{1}+\lambda_{2}ln{\dot{\varepsilon }}^{*}\right)\left(T-T_{r}\right)]$	[109]
Aluminum alloy (AA) 6016-T6	Integrated the strain rate hardening coefficient with the strain rate.	$\sigma =\left\{\lambda \left[A(\varepsilon_{0}+{\bar{\varepsilon }}^{p}{)}^{n}\right]+\left(1-\lambda \right)[Q_{s}-(Q_{s}-Q_{0})e^{\left(-\frac{V_{0}}{Q_{s}}{\bar{\varepsilon }}^{p}\right)}]\right\}\left\{1+\left[C_{0}+C_{1}ln\left(\frac{{\dot{\bar{\varepsilon }}}^{p}}{{\dot{\bar{\varepsilon_{0}}}}^{p}}\right)+C_{2}{ln}^{2}(\frac{{\dot{\bar{\varepsilon }}}^{p}}{{\dot{\bar{\varepsilon_{0}}}}^{p}})\right]ln(\frac{{\dot{\bar{\varepsilon }}}^{p}}{{\dot{\bar{\varepsilon_{0}}}}^{p}})\right\}$	[110]
2139-T8 aluminum alloy	Coupled the temperature and strain hardening terms, while incorporating temperature effects into the strain rate sensitive part.	$\sigma =\left\{A+B_{0}[1-(\frac{T-T_{0}}{T_{m}-T_{0}}{)}^{p}]\varepsilon^{n}\right\}\left[1+\left(c_{1}T_{r}^{*p}+c_{2}H\right)ln\frac{\dot{\varepsilon }}{{\dot{\varepsilon }}_{0}}\right][1-(\frac{T-T_{0}}{T_{m}-T_{0}}{)}^{p}]$	[111]
Si_p_/Al composites	Added thermal softening and damage terms to J-C model.	$\sigma =\left(A+B\varepsilon^{n}\right)\left(1+Cln{\dot{\varepsilon }}^{*}\right)\{aexp[-\left(\frac{T^{*}-m_{1}}{m_{2}}{)}^{2}\right]\}\{D+(1-D)[tanh(\frac{1}{\frac{\varepsilon_{cr}}{\varepsilon_{c}}\varepsilon }{)}^{r}{]}^{s}$}	[98]
Be/2024Al composites	Considered the coupling effect between hot processing parameters, strain, strain rate, and temperature.	$\sigma =\left(A+B_{1}\varepsilon +B_{2}\varepsilon^{2}+B_{3}\varepsilon^{3}\right)\left(1+C_{1}ln{\dot{\varepsilon }}^{*}\right)exp[\left(\lambda_{1}+\lambda_{2}ln{\dot{\varepsilon }}^{*}\right)T^{*}]$	[112]
B_4_C/Al composites	Introduced a decreasing function of strain to describe the softening phenomenon of the material.	$\sigma =(A+B\varepsilon^{n})(1-\varepsilon^{k})(1+Cln{\dot{\varepsilon }}^{*})(1-T^{*m})$	[74]
SiC_p_/Al composites	Transformed the thermal softening term in the original equation into terms related to strain rate and strain.	$\sigma =\left(A+B\varepsilon^{n}\right)\left(1+Cln{\dot{\varepsilon }}^{*}\right)\left[1-\left(\frac{\dot{\varepsilon }-1}{\left|\dot{\varepsilon }-1\right|}\right)k\varepsilon \right]$	[64]
aluminum matrix composites	Combined the L-R model and J-C model.	$\sigma =\left(A+B\varepsilon^{n}\right)g\left(f\right)\left(1+Cln{\dot{\varepsilon }}^{*}\right)[1+CV(f)ln{\dot{\varepsilon }}^{*}](1-T^{*m})$	[113]
Si_p_/Al composites	Introduced tangent function and Gaussian function to correct the effects brought by particle damage and thermal softening.	$\sigma =\left(A+B\varepsilon^{n}\right)\left(1+Cln{\dot{\varepsilon }}^{*}\right)\{aexp[-\left(\frac{T^{*}-m_{1}}{m_{2}}{)}^{2}\right]\}\{D+(1-D)[\tanh\left(\frac{1}{\frac{\varepsilon_{cr}}{\varepsilon_{c}}\varepsilon }{)}^{r}{]}^{s}\right\}$	[98]
TiB_2_/Al composites	The material constants B, C, and m in the original J-C model were determined using polynomial fitting.	$\sigma =\left(A+e^{f\left(\varepsilon \right)}\right)[1+C_{1}ln{\dot{\varepsilon }}^{*}+C_{2}(ln{\dot{\varepsilon }}^{*}{)}^{2}][1-(T^{*}{)}^{m\left({\dot{\varepsilon }}^{*}\right)}]$	[114]

## Data Availability

Data are contained within the article.
